# Reality check: where women look when viewing fashion magazine advertisements with disclaimer labels

**DOI:** 10.1186/2050-2974-1-S1-O53

**Published:** 2013-11-14

**Authors:** Belinda Bury, Marika Tiggemann, Amy Slater

**Affiliations:** 1 Flinders University, Australia

## 

It is now well documented that exposure to the thin ideal can negatively impact women's body image. One recent recommendation (National Advisory Group on Body Image, 2009) is that warning labels should be used to indicate when media images have been digitally altered. Thus far, preliminary research findings have been mixed as to the effectiveness of this strategy. The aim of the present study was to use eye tracking technology to examine where women look in fashion magazine advertisements when warning labels are affixed. Sixty female participants were allocated to one of three conditions: no warning label, a generic warning label, or a warning label referring to specific body parts. Significantly greater fixations (number, time) on the label area were found in the two label conditions, indicating that women did notice and attend to the label. There was, however, no difference in fixations on any specific body area. Nevertheless, in the specific label condition, the eye tracking recordings indicated that reading the label directed women's attention to the specified body area (examples to be presented). It was concluded that eye tracking may provide a useful methodology for examining attentional mechanisms underlying responses to media images and proposed interventions.

This abstract was presented in the **Body Image** stream of the 2013 ANZAED Conference.

